# Development and Implementation of a Registry of Patients Attending Multidisciplinary Pain Treatment Clinics: The Quebec Pain Registry

**DOI:** 10.1155/2017/8123812

**Published:** 2017-02-09

**Authors:** M. Choinière, M. A. Ware, M. G. Pagé, A. Lacasse, H. Lanctôt, N. Beaudet, A. Boulanger, P. Bourgault, C. Cloutier, L. Coupal, Y. De Koninck, D. Dion, P. Dolbec, L. Germain, V. Martin, P. Sarret, Y. Shir, M.-C. Taillefer, B. Tousignant, A. Trépanier, R. Truchon

**Affiliations:** ^1^Centre de Recherche du Centre Hospitalier de l'Université de Montréal (CRCHUM), Montréal, QC, Canada; ^2^Research Center of the Montreal Heart Institute, Montreal, QC, Canada; ^3^Department of Anesthesiology, Faculty of Medicine, Université de Montréal, Montreal, QC, Canada; ^4^Quebec Pain Research Network, Sherbrooke, QC, Canada; ^5^Alan Edwards Centre for Research on Pain, McGill University, Montreal, QC, Canada; ^6^Department of Family Medicine, Faculty of Medicine, McGill University, Montreal, QC, Canada; ^7^Department of Anesthesiology, Faculty of Medicine, McGill University, Montreal, QC, Canada; ^8^Département des Sciences de la Santé, Université du Québec en Abitibi-Témiscamingue, Rouyn-Noranda, QC, Canada; ^9^Department of Anesthesiology, Faculty of Medicine and Health Sciences, Université de Sherbrooke, Sherbrooke, QC, Canada; ^10^Pain Centre of Expertise, Integrated Health Network of Montreal University, Montreal, QC, Canada; ^11^Pain Clinic, Centre Hospitalier de l'Université de Montréal, Montréal, QC, Canada; ^12^School of Nursing, Faculty of Medicine and Health Sciences, Université de Sherbrooke, Sherbrooke, QC, Canada; ^13^Pain Centre of Expertise, Integrated Health Network of Sherbrooke University, Sherbrooke, QC, Canada; ^14^Department of Neurosurgery, Faculty of Medicine and Health Sciences, Université de Sherbrooke, Sherbrooke, QC, Canada; ^15^Pain Clinic, Centre Hospitalier de l'Université de Sherbrooke, Sherbrooke, QC, Canada; ^16^Impacts Inc., Saint-Lambert, QC, Canada; ^17^Quebec Mental Health Institute of Research Center, Quebec City, QC, Canada; ^18^Department of Psychiatry & Neurosciences, Faculty of Medicine, Université Laval, Quebec City, QC, Canada; ^19^Department of Family Medicine, Faculty of Medicine, Université de Montréal, Montreal, QC, Canada; ^20^Department of Anesthesiology, Faculty of Medicine, Université Laval, Quebec City, QC, Canada; ^21^Pain Clinic, Hôtel-Dieu de Lévis, Lévis, QC, Canada; ^22^Department of Pharmacology and Physiology, Faculty of Medicine and Health Sciences, Université de Sherbrooke, Sherbrooke, QC, Canada; ^23^Pain Centre of Expertise, Integrated Health Network of Laval University, Quebec City, QC, Canada; ^24^Pain Clinic, Centre Hospitalier Universitaire de Québec, Quebec City, QC, Canada

## Abstract

The Quebec Pain Registry (QPR) is a large research database of patients suffering from various chronic pain (CP) syndromes who were referred to one of five tertiary care centres in the province of Quebec (Canada). Patients were monitored using common demographics, identical clinical descriptors, and uniform validated outcomes. This paper describes the development, implementation, and research potential of the QPR. Between 2008 and 2013, 6902 patients were enrolled in the QPR, and data were collected prior to their first visit at the pain clinic and six months later. More than 90% of them (mean age ± SD: 52.76 ± 4.60, females: 59.1%) consented that their QPR data be used for research purposes. The results suggest that, compared to patients with serious chronic medical disorders, CP patients referred to tertiary care clinics are more severely impaired in multiple domains including emotional and physical functioning. The QPR is also a powerful and comprehensive tool for conducting research in a “real-world” context with 27 observational studies and satellite research projects which have been completed or are underway. It contains data on the clinical evolution of thousands of patients and provides the opportunity of answering important research questions on various aspects of CP (or specific pain syndromes) and its management.

## 1. Introduction

In the field of pain research, like in other medical fields, randomized controlled trials (RCTs) are the gold standard for establishing the efficacy of interventions. However, RCTs have several limitations [1–8]. Typically, patients are selected according to strict criteria, and the interventions are assessed under highly controlled conditions such that the obtained results are often poorly generalizable to everyday practices. Furthermore, RCTs are usually limited in time and sample sizes may be too small to detect serious adverse effects. In order to fill in these critical gaps in evidence for establishing best practices in pain management, patient registries and other forms of electronic healthcare databases represent valuable options [1, 3, 4, 6].

A patient registry is defined as “an organized system that uses observational study methods to collect uniform data (clinical and other) to evaluate specified outcomes for a population defined by a particular disease, condition or exposure, and that serves a predetermined scientific, clinical or policy purpose(s)” [2]. Patient registries contain “real-world” data generated during the course of patient care that can complement RCT findings. They can provide valuable information for determining the clinical effectiveness and safety of interventions when used in a diverse array of patients (e.g., variable age, multiple comorbidities) and clinical settings. Patient registries can also be designed to (1) describe the progression of diseases, (2) monitor quality of care, (3) assess the cost-effectiveness of treatments, or (4) conduct outcome research studies [1–3]. Although they also have their limitations [1, 2, 4], patient registries represent interesting and alternative research avenues and are becoming more and more popular in subspecialities of pain medicine including management of acute postoperative pain (e.g., [9]), rheumatic diseases [10, 11], low back pain (e.g., [12]), and neuropathic pain (e.g., [13, 14]) as well as pain rehabilitation (e.g., [15]) and military-specific pain services (e.g., [16] to name just a few.

In 2008, the Health Ministry of the province of Quebec (Canada) designated four Pain Centres of Expertise within the Montreal, McGill, Sherbrooke, and Laval University Health Networks which altogether cover the entire province. The Ministry wished to monitor the clinical outcomes of patients treated in these newly designated centres (and especially in tertiary care pain clinics) and obtain relevant administrative statistics. In parallel, one of the strategic plans of the Quebec Pain Research Network for 2007–2011 was to develop a province-wide clinical pain research infrastructure to facilitate the conduct of large observational and clinical studies. To meet the objectives of both of these organizations, there was a need to develop a uniform multisite registry that documents the clinical condition and evolution of patients treated in tertiary care pain clinics. This gave the impetus to implement in the Quebec Pain Registry (QPR) project designed to serve both clinical/administrative and research purposes. To our knowledge, only two other registries of patients with various types of chronic pain disorders treated in multidisciplinary clinics have been developed so far, one in the UK (Pain Audit Collection System) [17] (PACS) and one in the US (Collaborative Health Outcomes Information Registry (CHOIR) [18]). However, their data collection procedures differ from those used in the QPR whose content is also richer in terms of clinical/medical data and outcome measures.

The present paper describes how the QPR was developed and implemented detailing its strength and shortcomings with the aim of facilitating the creation of other pain patient registries. The QPR structure and content are also presented along with the characteristics of the enrolled patients. The policy and procedures for accessing QPR data sets for research purposes are described as well as the type of access requests made.

## 2. Methods

### 2.1. Aims of the QPR

The aims of the QPR project were to (1) put in place a prospective web-based registry of ambulatory patients suffering from various types of pain syndromes who were referred for multidisciplinary treatment in large university-affiliated pain clinics in the province of Quebec, (2) assess and monitor their condition over time using common demographics, identical clinical descriptors, and uniform outcomes measured with standardized/validated measurement tools in each participating site, (3) document pain treatments patients received and/or used over time, (4) provide clinicians with a summary of the individual condition of their patients along with useful administrative statistics for their pain treatment facility, and (5) provide reliable “real-world” data to researchers wishing to answer important research questions or test hypotheses regarding various aspects of chronic pain (or specific pain syndromes) and its management, to assess study feasibility, and to facilitate and speed up patient recruitment in research projects or clinical trials.

### 2.2. Development and Implementation

Using the guidelines proposed by Solomon et al. (1991) [19] and Gliklich and Dreyer (2007) [20], the development and implementation of the QPR involved two distinct phases.

#### 2.2.1. Phase I: Choice of the Variables/Outcomes/Measurement Tools and Pilot Study

The choice of items to be included in the QPR was made with the objective of creating a uniform* minimal needs-based data set*. The item choice had to be balanced between the clinicians' and researchers' interests for large amount of data, the burden placed on the patients, and the time/costs associated with the data collection process.

*Demographic and Clinical Variables*. All medical directors of large Canadian university-affiliated pain treatment clinics were contacted to share the questionnaires they used to record patients' demographics and clinical data (e.g., types of current and past pharmacological and nonpharmacological treatments received, drug adverse effects, and comorbidities) at the first visit in their facility and at follow-up time(s). These questionnaires were carefully reviewed by one researcher (M.C.), one pain clinician (D.D.), and a research nurse coordinator (H.L.) who selected the items to be included in the QPR based on recurrence of their appearance across questionnaires along with what they considered as the most optimal question formulation and categories of responses to measure these variables. Canadian and Quebec governmental health surveys (Statistics Canada [21],* Institut de la Statistique du Québec* [22]) were also reviewed to ensure uniformity with their coding system whenever possible (e.g., ethnicity, civil status).

All the above information except for patient sociodemographics was incorporated into a single questionnaire named the Initial Nurse-administered Questionnaire. A second questionnaire was also developed using the approach described above in order to collect follow-up data after the patients' first visit at the pain clinic (6-Month Nurse-administered Questionnaire).

With regard to patient pain diagnoses to be established by the pain physicians at the participating sites, it was felt that the International Classification of Diseases (ICD-9 or 10 systems) [23, 24] did not provide precise diagnostic codes for pain syndromes while the use of the coding system of the International Association for the Study of Pain [25] was viewed as too complicated and not practical in real-life clinical settings. Therefore, four experienced pain physicians with background in anesthesiology or neurosurgery who have been working for more than 15 years in tertiary care pain clinics in the province of Quebec and who took part in the later phases of the QPR (A.B., C.C., P.D., and Y.S.) were invited to elaborate a comprehensive and consensual grid of pain diagnoses to which were assigned codes based on the location of the pain (e.g., thoracic pain, generalized pain syndrome), the type of disorder (e.g., postmastectomy pain, and fibromyalgia), and/or its suspected etiology (e.g., disc disorder, pain following chemotherapy/radiotherapy). The DN4 Questionnaire was also added to screen for the presence/absence of a neuropathic pain component [26]. In order to ensure uniformity in data collection for the type of medical interventions carried out at the pain clinic (e.g., blocks, epidural injections, and neurolysis), the clinicians elaborated a second grid which listed the possible interventions to which were assigned different codes.

*Patient Outcomes*. Different sources of information such as the recommendations made by the Initiative on Methods, Measurement, and Pain Assessment in Clinical Trials (IMMPACT) Group [27, 28] guided the choice of the core outcome domains and measurement tools to be included in the QPR. A comprehensive review of the scientific literature was also carried out by a postdoctoral fellow (M-C.T) under the supervision of M.C. in order to list the strengths and limitations of existing validated instruments in English and French language to measure (1) pain characteristics (e.g., intensity, interference), (2) emotional well-being, (3) health-related quality of life, (4) treatment expectations, and (5) perceived improvement and treatment satisfaction. Accessibility to normative data, respondent burden, and research experience gained through the multisite Canadian STOP-PAIN Project [29] were also factors that were considered in the selection of measurement tools. In the few cases where a validated French version of the English measurement tool was not available (e.g., Chronic Pain Sleep Inventory [30]), the items were translated into French using a forward-backward method of translation [31]. The questionnaires/scales selected through the above process were then assembled into two questionnaires named the Initial Patient self-administered Questionnaire and the 6-Month Patient self-administered Questionnaire. All the questionnaires were then distributed to the different members of the QPR team (clinicians and researchers) for a final round of comments.

Paper Case Report Forms (CRFs) were then prepared and used in a pilot prospective study to test the feasibility of implementing/running the QPR. Additional pieces of information were collected during the pilot study such as the time taken to complete each questionnaires/interview, patients' perceived degree of difficulty for filling out the questionnaires, and clinical usefulness of the collected material. Physicians were also asked to check the items they would like to be included in a clinical summary form. After having obtained institutional ethic approval of the research protocol, the pilot study was conducted in 2007-2008 with 90 consecutive patients recruited in three multidisciplinary pain treatment facilities which were candidates for becoming the designated tertiary care clinics of the Quebec Pain Centres of Expertise. These clinics were, respectively, located at the* Centre Hospitalier de l'Université de Montréal* (CHUM), McGill University Health Centre (MUHC), and* Centre Hospitalier de l'Université de Sherbrooke* (CHUS). Once patients provided informed consent, the QPR questionnaires were administered to them prior to their first visit at the pain clinic and six months later. All the data collected in this pilot study were analysed using descriptive statistics. The results of these analyses along with the comments/suggestions from the stakeholders (unpublished data) were summarized by the two principal investigators (PIs) of the QPR project (M.C., M.W.) along with D.D. and H.L. and were used to develop the final versions of the QPR questionnaires and clinical summary forms, adjust the procedures to maximize patients' responsiveness and retention at follow-up, and ensure optimal collaboration of the pain clinicians in the project.

*QPR Measures and Tools*.  Table 1 lists the demographic variables, clinical data, patient outcomes, and measurement tools used in the QPR along with some administrative data obtained from the patients' medical record (e.g., time elapsed between referral and first visit at the pain clinic). Information is also available on the QPR web site (http://www.quebecpainregistry.com). Several items contained in the original version of the registry were withdrawn at the end of June 2012 (see Table 1) to reduce staff costs. Based on the results of a survey carried out among the QPR users in the preceding months, it was felt relevant to add one instrument which was increasingly used to assess the risk of opioid abuse, that is, the Opioid Risk Tool [32, 33]. On the same occasion, the pain diagnostic grid and the medical intervention grid were reviewed to include additional codes. Copies of these grids are reproduced in the supplementary files (see supplementary files in Supplementary Material available online at https://doi.org/10.1155/2017/8123812) of the online version of the present article.

*Development of the Web-Based QPR Database*. Dacima Software Inc. (Montreal, Quebec, Canada; http://www.dacimasoftware.com) developed, tested, hosted, and maintained the central web-based quality-controlled QPR database which was FDA 21 CFR 11-compliant. They also developed the electronic CRFs and database-generated clinical summary forms which were beta tested by the Registry Nurse Coordinator (H.L.) and her assistant (L.G.). In April 2012, the QPR central database was transferred to Typhon Solutions Inc. (Montreal, Quebec, Canada; http://typhonsolutions.ca) who also developed and updated the electronic CRFs and clinical summary forms. System access controls were in place for registered users from the participating sites. Access to the central anonymized database was limited to authorized staff (e.g., biostatistician). Each patient was given a unique code number in the database which was not linked to her/his medical record. All the names, addresses, and phone numbers of the participants along with their unique code number were kept in each participating site in a separate and password secured Excel file which was accessible only to the RN and RA. This was aimed at preventing the transmission of specific patient identifiers in the central data repository.

#### 2.2.2. Phase II: Implementation, Data Collection, and Quality Monitoring

*Ethics Approval*. The protocol, questionnaires, and procedures for implementing the QPR in the multidisciplinary pain clinics of the CHUM, MUHC, and CHUS were approved in 2008 by the REB of the CHUM which acted as the central ethic committee in charge of obtaining approval from the local ones. Given that the designation of the tertiary care pain clinics affiliated to the Pain Center of Expertise of Laval University (Quebec City, Canada) (*Centre Hospitalier Universitaire de Québec *(CHUQ) and the* Hôtel-Dieu de Lévis* (HDL)) was considerably delayed for administrative reasons, ethic approval was obtained only in 2012 for implementation of the QPR in these two sites.

*Implementation of the QPR. *The QPR was implemented at the multidisciplinary pain treatment clinics of the CHUM, CHUS, and MUHC in November 2008, January 2009, and March 2009, respectively. At the HDL, the QPR project started in July 2012, and in August 2012 at the CHUQ. Due to a restructuring of the Pain Center of Expertise of the Sherbrooke University Health Network, enrolment of new patients in the QPR had to be stopped at the pain clinic of the CHUS in August 2012 but 6-month follow-up measures were collected up to February 2013. In each clinic, patient pain management was personalized and involved various types of pharmacological (e.g., opioids, antidepressants, and anticonvulsivants), interventional (e.g., nerve blocks, epidural injections), physical (e.g., physiotherapy, electrostimulation), and/or psychological (e.g., cognitive-behavioral therapy) modalities as well as teaching of self-management techniques (e.g., relaxation, distraction, and sleep hygiene). Patients could be seen by professionals from various disciplines at the pain clinic including anesthesiology, family medicine, neurology, nursing, pharmacy, physiatry, psychology, psychiatry, and physiotherapy. Choice of the treatment modalities was based on the patients' needs and varied from one to the other.

*Patient Enrolment*. Consecutive ambulatory patients scheduled for a first visit at the pain clinic for multidisciplinary pain treatment considerations were enrolled in the QPR if they were (1) aged ≥ 18 years and (2) able to understand and read French or English. Patients who were unable to complete questionnaires due to severe physical or cognitive inability were excluded. Patients who were eligible for enrolment in the preexisting registry of fibromyalgia patients [34] at the MUHC pain clinic only were also excluded. Eligible patients were informed that the information collected with the QPR questionnaires before their first appointment and at follow-up(s) was needed for clinical purposes (production of a summary of their clinical condition for the physician with whom they had an appointment) and administrative endeavors (production of annual anonymized statistics). Patients were also informed that their data along with those of other patients who gave their permission could be used for research purposes. If they agreed, they were invited to sign the REB-approved consent form of the QPR.

*Data Collection Procedures*. Once the patients' first appointment was fixed, the receptionist of the pain clinic faxed their contact information to the Registry Assistant (RA) who contacted them to explain the QPR procedures and confirm their eligibility. She informed them that they would receive by mail the Initial Patient Questionnaire along with the QPR consent form and a preaddressed/stamped envelope. At the time the QPR was implemented in 2008, e-technologies such as IPad, IPhone, and other android devices were not widely spread in the province and a substantial number of patients did not have access to Internet yet. For those who did, they were sent by e-mail an electronic copy of the Patient Questionnaire that they completed on screen. Patients were told that completion of the questionnaire would require 20–30 minutes of their time and that the Registry Nurse will contact them by phone prior to their first visit at the pain clinic. Upon reception of the questionnaire, the RA carefully reviewed it to make sure that all questions had been answered and if not, the patient was contacted by phone. If the questionnaire was not returned within the week preceding the scheduled appointment at the pain clinic, the RA phoned the patient and asked to bring it on the day of her/his appointment at the pain clinic. Data collected with the Initial Patient Questionnaire were entered by the RA into the web-based QPR portal.

The RA contacted the Registry Nurse (RN) who conducted a structured telephone or face-to-face interview with the patients in the days/hours preceding their first appointment at the pain clinic using the Initial Nurse Questionnaire. Depending on the patient's clinical condition, the interview lasted between 30 and 90 minutes, and the information was entered by the RN or RA into the online QPR database. A summary of the patient's clinical condition (e.g., pain duration and intensity, and analgesic intake) was then generated from the database and transmitted to the treating physician of the pain clinic. Follow-up data were collected using the 6-Month Patient and Nurse Questionnaires using a similar methodology as the one described above. Additional follow-up data were gathered at 12 and 24 months after the initial visit but only in those patients who had not been discharged from the pain clinic in the meantime. These data were collected with questionnaires containing the same measures as those administered at the 6-month follow-up. Due to financial considerations, follow-up data were not collected at 12 and 24 months in newly enrolled patients after March 2012. Due to budget cuts in the QPR project for the year 2014-2015, collection of 6-month follow-up data had to be interrupted in newly registered patients after June 2014, and enrolment of new patients ended in November 2014.

In order to have a more complete picture of patient pain management than the one provided in medical records, all data about pharmacotherapy (prescribed and over-the-counter medication) and nonpharmacological treatments used inside and outside of the pain clinic (including complementary alternative therapy) were collected by the Registry Nurses who did not have clinical duties in the QPR participating sites. The purpose of having nurses rather than RA for conducting the interviews with the patients was twofold: (1) to ensure accuracy of the patient clinical summary transmitted to the treating physician at the pain clinic and (2) to optimize the quality of the medical/clinical data contained in the QPR database.

*Quality Safeguards*. Standard operating procedures (SOPs) were prepared to standardize patient enrolment and data collection, entry, and quality. Training of the QPR staff was under the responsibility of the Registry Nurse Coordinator and her assistant and consisted of a 2-day meeting during which the SOPs were carefully reviewed, explained, and illustrated with examples and mock patient interviews. Phone and e-mail follow-ups were made to maintain staff competency, inform them about modifications in the SOPs, and answer questions. Onsite audits were also carried out in the participating clinics to review screening/follow-up logs and ensure procedural consistency across sites. Finally, all the QPR staff attended a face-to-face meeting with the PIs and the Coordinator at least once a year to monitor QPR progress, review the SOPs, and reiterate the high importance of data completeness.

Quality monitoring of the QPR database was under the responsibility of the Registry Nurse Coordinator and her assistant. Each participating site was requested to provide a monthly report of the number of patients enrolled in the registry, reasons for exclusion, number of questionnaires not completed and reasons why, losses to follow-up, and so on. With regard to data quality monitoring, a series of quality controls were programmed in the QPR database to allow instant automated data validation checks (e.g., out-of-range values, logical inconsistencies). To facilitate medication data entry and ensure consistency (e.g., generic versus brand name), a medication dictionary was built in the database. Manual data cleaning was also carried out on a regular basis to identify discrepancies and missing data on variables targeted as important (e.g., patient diagnosis, pain duration, medication, and medical history) and to generate “queries” to be sent to the participating sites for resolution. Statistical programs to identify errors or inconsistencies on specific measures were also part of quality control activities. Errors identified when data were analysed for administrative or research purposes were also corrected in the database.

### 2.3. Access Policy to QPR Data and Business Model

*Access Policy*. Once the QPR was implemented, a comprehensive policy to access/use data from QPR patients contained in the registry and a business cost-recovery model were developed by the two PIs of the present project (M.C., M.W.) in collaboration with members of the Executive Committee of the Quebec Pain Research Network (Y.D.K., P.S., and N.B.) and legal/administrative advisers. The data access policy received ethical approval from the central REB for the QPR project at the CHUM and is available in the supplementary files of the online version of the present article and on the QPR website (http://www.quebecpainregistry.com).

Datasets of patients who gave informed consent can be accessed for conducting observational studies. Assessment of feasibility of research projects or clinical trials is also possible (e.g., number of QPR female patients aged between 30 and 50 years with a diagnosis of complex regional pain syndrome). The QPR can also be used to conduct “satellite” research projects, that is, studies in which data contained in the QPR (e.g., age, sex, and types of pain medication) are linked to other sets of data (e.g., governmental administrative databases, data collected in the context of a new study on variables other than the ones contained in the QPR). However, the research protocol and the accompanying patient consent form of the satellite projects have to receive prior approval by the clinical team of the participating site(s), the central REB of the CHUM, and the local REBs. Once the project is approved by these authorities, the Medical Director of the participating clinic(s) sends a letter to inform the eligible QPR patients about the research project and invites them if they are interested in participating to contact the person in charge of the study or her/his representative. Finally, the QPR data can be accessed to facilitate and speed up patients' recruitment in research projects or clinical trials; the procedure is the same as the one used for satellite research projects.

*Business Model*. The registry is an academic, not-for-profit project. The business model relies on fees to cover (1) administrative costs for running the data access requests based on their complexity level (e.g., preparation of the extraction/analysis plan, data extraction, statistical analyses, and report preparation) and (2) financial contribution to maintain the QPR data repository and ensure its long-term sustainability. Access fees vary according to the type of requesters, the lowest costs being for academic researchers who are members of the Quebec Pain Research Network (QPRN), followed by academic researchers who are not part of the QPRN, and industry researchers whose companies were or were not funding partners of the QPRN. Fees for accessing QPR data also vary as a function of the number of variables requested, complexity of the extraction process and statistical analysis (if applicable), and whether they have to be linked or not to external data sets (e.g., governmental administrative database).

### 2.4. Analyses of the QPR Data

The total number of patients enrolled in the QPR between November 1, 2008, and December 21, 2014, is 9363 (http://www.quebecpainregistry.com) but the data included in the present article cover the period during which new patients were enrolled in the QPR up to December 31, 2013, and followed up at 6 months until to June 30, 2014. Patients who did not give consent for their QPR data to be used for research purposes were excluded from the analyses. Data describing the clinical evolution of the subgroup of patients with follow-up data not only at 6 but also at 12 and 24 months have been presented at the Annual Scientific Meeting of the Canadian Pain Society in 2014 [35] and are in the process of being submitted for publication.

#### 2.4.1. Missing Questionnaires

The number and percentages of the Patient and Nurse Questionnaires which were completed prior to the initial visit at the pain clinic and at 6-month follow-up were computed. In order to assess if missing questionnaires qualified as “missing at random” [2, 36], differences between completed questionnaires and missing ones at each time point and between time points were examined according to patients' age using independent Student *t*-tests. Chi-squared tests were carried out on differences between sex and participating pain clinics (study site). However, such significant testing in studies involving large sample sizes like the present one can be misleading because even small differences can reach statistical significance while they can be viewed as trivial and not meaningful clinically [2, 37, 38]. Therefore, effect sizes of age differences between patients who completed and did not complete the Patient or Nurse Questionnaires at each time point were calculated with Cohen's *d* [39]. Only differences which reached a medium to large size effect as defined by Cohen [39] (i.e., a *d* value ≥ ±0.5) were considered meaningful [38]. For the variables sex and study site, effect sizes were calculated using, respectively, the Phi (*φ*) [40] and Cramér's *V* [41] statistics, and only those which were in the moderate to strong range (i.e., a *φ* or Cramér's *V* value ≥ ±0.3) were judged as being clinically important [2, 38].

#### 2.4.2. Patients' Characteristics

Descriptive statistics including measures of central tendency (mean or median) and dispersion (standard deviation or range) along with frequency tables were used to document the characteristics of the patients enrolled in the QPR. Due to space limitation, only a subset of the variables in Table 1 which were believed to provide a broad profile of the QPR patients at the time of their first visit at the pain clinic were analysed in the present article along with some data collected at 6-month follow-up. Student's *t*-tests were used to compare mean scores obtained on the physical and mental summary scales of the SF-12v2 [42, 43] in QPR patients at their first visit at the pain clinic to those of (1) the US healthy population (Canadian data being currently unavailable) and (2) patients suffering from serious chronic medical disorders other than chronic pain (cancer, heart disease, and diabetes) [42].

#### 2.4.3. Requests for Access to QPR Data

Descriptive statistics were computed on the kind and number of requests made for accessing QPR data and the type of users.

## 3. Results

### 3.1. Recruitment and Record Completeness

**Figure 1:**
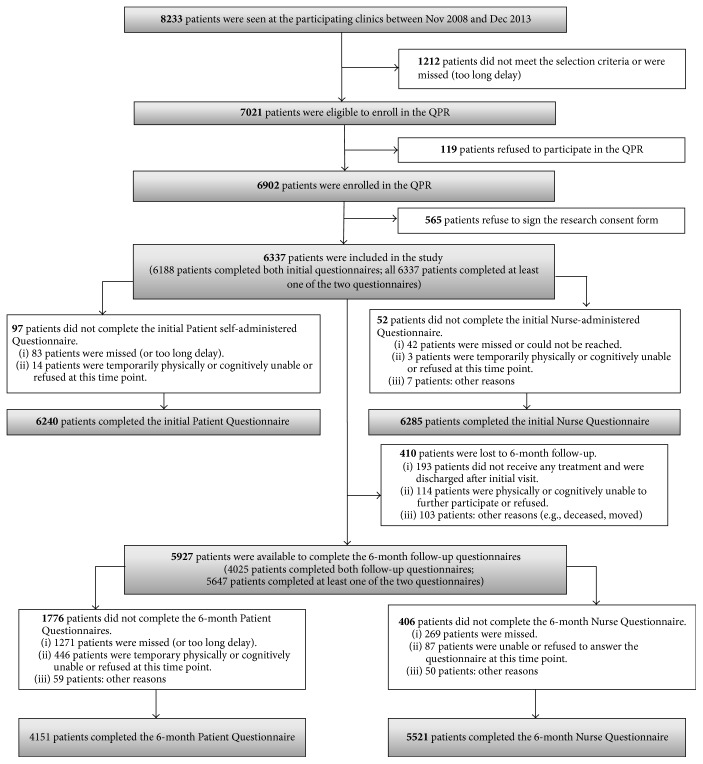
Flow of participants through the QPR during the study period.

Of the 8,233 patients who were referred to the participating pain clinics between November 2008 and December 2013 (inclusively), 7021 (85.3%) qualified for enrolment in the QPR and only 1.7% refused to do so (Figure 1). Ninety-two percent (6337/6902) consented that their QPR data be used for research purposes. Given that the registry was implemented in each participating site at different moments, the patient distribution was variable between sites: CHUM: *N* = 2052 (32.4%); MUHC: *N* = 2292 (36.2%); CHUS: *N* = 745 (11.8%); CHUQ: *N* = 810 (12.8%); HDL: *N* = 438 (6.9%). The percentages of patients who completed the Initial Patient and Nurse Questionnaires were 98.5% and 99.2%, respectively. At 6-month follow-up, 89.1% of the patients (5647/6337) completed at least one of the two questionnaires (Figure 1). Results of the statistical analysis for comparing participants who completed and did not complete the questionnaires at each time point and between time points revealed some statistically significant differences with regard to age, sex, and/or study site (*P* ≤ 0.05). However, all the effect sizes were small (*d* values < 0.5; *φ* or Cramér's *V* values < 0.3) suggesting that the differences are not clinically meaningful [2, 38].

### 3.2. Characteristics of the QPR Patients

Patients enrolled in the QPR during the study period were aged between 18 and 88 years (mean = 52.76, SD = 14.6), 59.1% were female, and the vast majority (92.0%) were of Caucasian origin (Table 2). Nearly half of them (46.1%) had a secondary level of education or less. The percentage of patients who worked on a part-time or full-time basis was 28.1% while permanent or temporary disability income was the unique source of revenue for more than one-third of the patients (37.2%).

The median amount of time elapsed between patients' referral and first visit at the pain clinic was 3.5 months; 35.0% of the patients waited more than 6 months for their appointment, some of them (5.2%) having waited between 2 and 4 years (Table 3). Close to 40% of the patients (38.7%) were referred at the pain clinic by their family physician while the others by specialists from various surgical (e.g., orthopedics, neurosurgery plastic surgery) or medical (e.g., neurology, rheumatology, and physiatry) disciplines (data not shown). Pain was present for 5 years or more in close to half of the patients (46.6%) and less than 1 year in 13.1% (Table 3). Since the onset of their pain, patients reported having consulted between 1 and 23 different types of healthcare professionals in medical (e.g., family medicine), physical (e.g., physical therapy), counseling (e.g., psychology), and/or alternative disciplines (e.g., acupuncture), the median value being 5.0 (data not shown).

**Figure 2:**
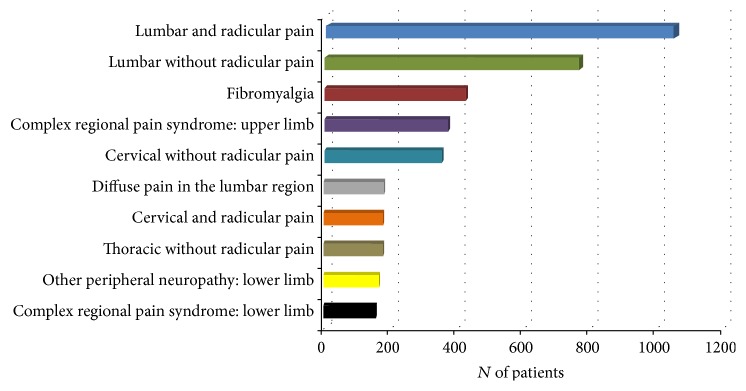
Top 10 pain diagnoses made by the physicians of the pain clinics.

An accident or a trauma was at the origin of the pain in more than half of the cases (52.5%); 31.4% of the patients reported that their pain occurred during or following an illness or and 14.2% after a surgery while one patient out of five (22.5%) was unable to associate the onset of her/his their pain to any precise event.  Figure 2 shows the top 10 pain diagnoses made by the physicians at the participating pain clinics. Lumbar pain with and without radicular pain was the most frequent one (28.6%), followed by fibromyalgia (6.6%), and complex regional pain syndrome in the upper limbs (5.7%). Based on both the clinicians' pain diagnoses and scores ≥ 4 obtained in the patient and physician portions of the DN4 [26], 31.0% of the patients were suffering from a neuropathic type of pain while the evidence was mixed in 45.0% of cases (i.e., the clinician diagnosed the patient with a neuropathic pain disorder but the DN4 score was not ≥4, or vice-versa) (Table 3).

**Figure 3:**
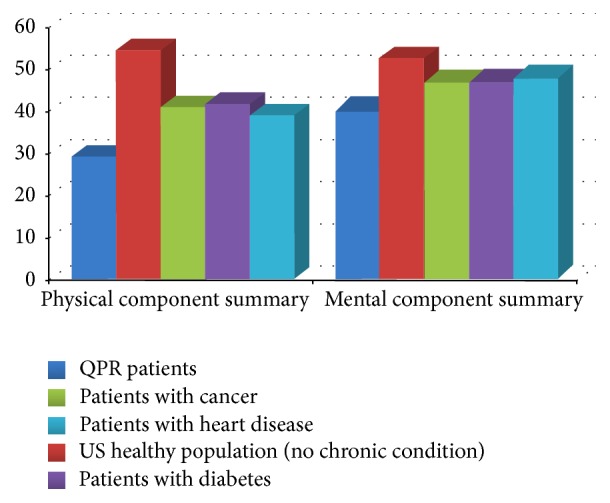
Mean scores on the physical and mental summary scales of the SF-12v2 in QPR patients (*n* = 6230), US healthy population without a chronic condition (*n* = 1275), and patients suffering from cancer (*n* = 246), diabetes (*n* = 530), and heart disease (*n* = 643) [42]. QPR patients' scores were compared to those of the other groups using Student's *t*-tests. All *P* values are < 0.001 and all Cohen's *d* values are ≥ 0.5.

The majority of the patients (85.0%) reported that their pain was present continuously in the 7 days preceding their first visit at the pain clinic. Mean pain intensity scores for the “average” pain and “worse” pain during this time period were 6.71 (SD = 2.0) and 8.16 (SD = 1.80), respectively. Patients' ratings on the interference scales of the Brief Pain Inventory-10 [44–46] revealed that, for more than 50% of them, pain severely impacted (scores ≥ 7/10) on various aspects of their daily living including general activity, normal work, sleep, and recreational and social activities (Table 3). A similar pattern of results emerged on reported health-related quality of life measured by the SF-12v2 [43]. The mean norm-based scores on the physical (29.07, SD = 8.90) and mental summary scales of this questionnaire (40.48, SD = 11.70) were significantly lower in our sample of patients suffering from chronic pain compared not only to those obtained in the US healthy population but also to patients suffering from serious medical chronic disorders (Figure 3) (all* P *≤ 0.001 and Cohen's *d* values between 0.6 and 3.3). With regard to depression symptomatology, scores obtained on the Beck Depression Inventory-I [47, 48] revealed signs of moderate to severe depression in 43.9% of the QPR patients (Table 3).

When questioned about the expected percentage of pain relief at six months after initiating treatment at the pain clinic, more than half of the patients (53.4%) anticipated pain relief ranging between 50 to 80% while one patient out of four (25.0%) expected pain relief superior to 80%. A large percentage of patients also anticipated that their functioning level (63.4% of patients) and quality of life (65.3% of patients) would be greatly or considerably improved over the next six months.

### 3.3. Requests for Access to QPR Data

Table 4 shows the number and type of research projects for which access to QPR data has been requested up to February 2016. Of the 40 projects, one-half were or are currently conducted by graduate students and post doc or medical fellows from various disciplines including anesthesiology, biomedical sciences, family medicine, neurosciences, pharmacology, psychology, and rehabilitation. Most of them consisted of observational studies or satellite research projects on various aspects of chronic pain or its management. The results of seven of these studies have been published so far [49–55] or are under review in peer-reviewed journals while others have been presented in scientific meetings [35, 56] and are in the process of being submitted for publication. Finally, several academic or industry researchers requested access to QPR data to either conduct studies or assess trial feasibility (Table 4).

## 4. Discussion

As shown in the present paper, developing and implementing a multisite patient registry is a complex task. Although maintaining a registry such as the QPR is very costly, we have shown that it is feasible to collect uniform and reliable data in a large number of tertiary care patients suffering from a variety of pain syndromes and across different clinics. The collected information can help clinicians in making their diagnosis and management plan and can provide participating pain clinics with useful statistics on their practices. In addition to documenting the characteristics and management of patients referred to multidisciplinary pain clinics, our results showed that the QPR made possible the conduct of observational studies and satellite research projects using “real-world” data on various aspects of chronic pain.

In terms of the feasibility of implementing the QPR, our results showed that only 1.7% of the potentially eligible patients refused to complete any questionnaires and 8.2% did not consent that their data be used for research purposes. Close to 100% of patients completed both the Initial Patient Questionnaire and Nurse Questionnaire. At 6-month follow-up, the percentage decreased but the overall retention rate remained high; that is, nearly 90% completed at least one of the two questionnaires. Some statistically significant differences were found in terms of patients' age, sex, and study site between those who did and did not answer the questionnaires at each time point and between time points. However, all effect sizes were not clinically meaningful suggesting that missing questionnaires did not introduce bias in the QPR data [2, 38].

Compared to the majority of existing pain patient registries [9–16], the QPR is somewhat unique in that it covers a wide variety of chronic pain disorders. Based on our literature review, there are only two other longitudinal registries which have been implemented in multidisciplinary pain treatment clinics, that is, the PACS [17] (also named PainDB [57]) and the CHOIR [18]. However, a validation study on the quality of the PainDB concluded that this registry was unsuitable for research purposes [57]. Implemented in 2012, that is, four years after the QPR, the CHOIR (https://choir.stanford.edu) [18] had several advantages including (1) extensive use of e-technologies (web, IPad, and IPhone/android devices) and (2) integration of item banks drawn from the National Institutes of Health (NIH) Patient-Reported Outcomes Measurement Information Systems (PROMIS) [58] which are administered using an item-response theory approach [59, 60]. The outcome measures included in the CHOIR are also collected using state-of-the-art computerized adaptive testing (CAT) techniques [61, 62] allowing the identification of the optimal items within each domain based on prior responses from the patients thereby reducing respondent burden [18]. As a result, the CHOIR collection of patient-reported outcomes is entirely electronic but more importantly the whole process is much more sophisticated and efficient in the CHOIR than it was in the QPR. The CHOIR outcome data can be linked to electronic medical records (EMR) thereby offering the possibility of point of care reporting to support clinical decisions as well as the opportunity to conduct multisite treatment effectiveness studies in a “real-world” context as opposed to the strict and artificial conditions of a RCT [18]. However, carrying out such a type of studies is also possible with the QPR as illustrated in earlier QPR publications on gabapentin on- and off-label use [50] and effectiveness of long-term opioid therapy [63]. Furthermore, the patients' pain diagnoses contained in the QPR are much more precise as they were established by pain specialist physicians using a comprehensive grid rather than being based on referral reason(s) or ICD-9 codes as it is the case in the CHOIR studies [64, 65]. This is a major advantage given the potential inaccuracy of diagnoses on the referral form. For example, it has been found that only 34% of patients referred for fibromyalgia actually do suffer from this disorder [66]. A last advantage of the QPR is that it contains data on pain modalities used by the patients inside and outside of the pain clinic (e.g., over-the-counter medication, complementary and alternative medicine therapies); those data were collected by registered nurses during comprehensive interviews. Although the QPR has several assets, they are also revealed to be its Achilles heel due to the huge associated costs in terms of human resources; those costs have compromised its long-term sustainability and expansion to other sites. In addition to the Registry Nurse Coordinator and her assistant, QPR data collection and data cleaning required at least one nurse and one administrative assistant working on a full-time basis at each participating site. In spite of the fact that we reduced the number of follow-up time points to only one (i.e., 6 months after patients' initial visit), the inclusion of two additional sites coupled to budget cuts in our funding forced us to stop enrolling new patients in the QPR although the database still continues to be available for research purposes. Enrolment of new patients was expected to be maintained in the long-term based solely on the revenues generated by data access requests from academic and industry researchers. However, these revenues are revealed to be insufficient. This may partly be the result of some delay in developing the mechanisms for accessing rapidly and efficiently QPR data and disseminating it to relevant audiences [19]. Other registries appeared to either have a more sustainable business model because of hospital membership fees and support from associations [9, 67] or have an overall lower maintenance cost due to the use of electronic data collection systems linked to ERM [18].

With regard to the characteristics of the participants enrolled in the QPR prior to their first visit at the pain clinic, our results highlight the fact that patients attending tertiary care pain clinics are significantly impaired in multiple domains. The majority of patients experienced continuous pain that reached intensity levels severe enough to interfere substantially with various aspects of their daily living including emotional well-being. Consistent with earlier results obtained in a smaller sample [29], we observed that patients reported poor health-related quality of life. The reported decrease in physical and mental functioning is remarkable when compared to the US healthy population and patients suffering from other chronic disorders [42]. However, these results are not that surprising when one considers that most patients are referred to tertiary care pain clinics once all other resources have been exhausted [29]. Since their pain onset, QPR patients reported having consulted up to more than 20 different types of healthcare professionals.

Interestingly, the median time elapsed between patients' referral and first visit at the pain clinic was found to be 3.5 months. Eleven years ago, Veillette et al. (2005) examined the waitlists of pain services across the province of Quebec and found that two-thirds of the patients were waiting for 9 months or more [68]. In a subsequent study, Peng et al. (2007) reported that the median wait time for a first appointment in the Quebec multidisciplinary pain treatment clinics was around 8 months [69]. In the light of the results obtained in the present study, it is tempting to speculate that local initiatives to improve patients' triaging [70] have contributed to somewhat decrease the patient waitlists of participating pain clinics.

In terms of expectations toward treatment, our results revealed that close to 70% of the patients anticipated great or considerable improvement in their functioning and quality of life while one patient out of five was expecting 80–100% pain relief following treatment at the pain clinic. Whether such high expectations can be detrimental to patients' outcomes was recently investigated using the initial visit and 6-month follow-up QPR data. The results of this study suggest that individuals who expected positive changes were more inclined to perceive improvements in their overall condition, leading to superior clinical outcomes [49].

Although our above study findings are informative and are based on a large sample size of patients followed prospectively in several sites and in a real-world context, they have limitations that should be acknowledged. First, they characterized only a small proportion of the chronic pain population, that is, those who are referred to tertiary care clinics, such that the results cannot be generalized to other populations of patients treated in primary or secondary care settings. Second, it is important to point out that access to tertiary care clinics in the province of Quebec requires a physician referral; access to these clinics is free but limited due to relatively long waiting lists as is the case in other Canadian provinces [69]. As a result, it is unclear how the data obtained in the QPR compare to what would be obtained in other healthcare systems (self-referrals or other systems of access to the specialized pain clinics). Finally, other limitations of our findings pertain to the use of an observational data source in which sampling and confounding biases may occur and thereby compromise validity of the conclusions [1, 2, 4]. Although we made all efforts to minimize missing questionnaires, we cannot exclude the possibility of biases.

The present paper finally illustrates how “real-world” patient registries such as the QPR can be valuable and powerful research tools [1, 2, 6]. So far, 21 observational studies on a variety of issues related to chronic pain have been carried out with QPR data or are underway. Six satellite research projects in which QPR data were interfaced with other databases or data sets have also been conducted, thereby minimizing duplicate data collection. As part of their research training, several graduate and postgraduate students used or are currently using QPR data to conduct research projects, and the results of seven of them have been published so far in peer-reviewed journals [49–55] or are under review.

## 5. Conclusions

The QPR is a vast registry of patients referred to multidisciplinary pain treatment clinics that was designed for clinical/administrative and research purposes. This registry provides numerous opportunities to study various aspects of chronic pain (or specific pain syndromes) and its management using longitudinal “real-world” data on a large set of variables collected in tertiary care patients. The most important challenge posed by the QPR remains to be its maintenance costs which have compromised its long-term sustainability and its expansion in other pain clinics.

## Supplementary Material

The first file shows the list of pain diagnoses and their corresponding codes contained in the Quebec Pain Registry (QPR). The pain physicians at the participating sites used this diagnostic grid to record the type(s) of pain the patients suffered from.
The second file shows the list of medical interventions and their corresponding codes in the QPR. This grid was used to record the type(s) of medical interventions the patients received.
The third file displays the policy to access and use data from the QPR.

## Figures and Tables

**Table 1:** Variables, outcomes, and measurement tools of the Quebec Pain Registry at each time point.

Variables/outcomes collected with the Patient self-administered Questionnaire (QP) and the Nurse-administered Questionnaire (NQ)	Initial visit	6-month follow-up^*∗*^
Pain history		
(i) NQ: pain duration	X	
(ii) NQ: circumstances surrounding the onset	X	
(iii) NQ: 1st degree family history of chronic pain^*ψ*^	X	
(iv) NQ: date and reason of referral, speciality of the referring doctor	X	
(v) NQ: number of pain-related visits to emergency (past 6 months)	X	X
(vi) NQ: number of pain-related hospitalizations (past 6 months)	X	X
(vii) NQ: time elapsed between consultation request and 1st visit at the Pain Clinic	X	
Pain characteristics		
(i) NQ: frequency in the past 7 days (always, occasionally, no pain)	X	X
(ii) PQ: intensity (pain now, average, and worst pain in the past 7 days) *(Numerical rating scale, 0 = no pain, 10 = worst possible pain)* [28]	X	X
(iii) NQ: quality (neuropathic pain component) *(DN4 Questionnaire)* [26]	X	X
(iv) PQ: pain interference on daily activities *(Interference Items of the Brief Pain Inventory-10)* [44–46]	X	X
(v) NQ: impact of pain on sleep *(Chronic Pain Sleep Inventory)* [30]	X	X
(vi) NQ: mobility support required inside and/or outside the home	X	X
(vii) NQ: pain diagnosi(e)s established at the pain clinic: location, type, suspected etiology	X	X
Psychological well-being and quality of life		
(i) PQ: depression *(Beck Depression Inventory-1)* [47, 48]	X	X
(ii) PQ: anger *(numerical rating scale*, *0 = not at all, 10 = extremely)*^*ψ*^	X	X
(iii) PQ: tendency to catastrophize in the face of pain *(Pain Catastrophizing Scale)* [71, 72]	X	X
(iv) PQ: health-related quality of life *(SF-12v2)* [42, 43]	X	X
Pain treatments at the pain clinic *or* elsewhere		
(i) NQ: current pharmacological pain treatment (prescribed and not prescribed): medication name and posology	X	X
(ii) NQ: side effects of current pharmacological pain treatment: type and severity *(categorical rating scale, 0 = none, 4 = severe)*^*ψ*^	X	X
(iii) NQ: past pharmacological pain treatment (prescribed and not prescribed): medication name and reason(s) for stopping	X	X
(iv) NQ: type of current and past nonpharmacological pain treatments including interventions (e.g., injection therapy, surgery), psychological techniques (e.g., self-management program, individual psychotherapy), self-management strategies (e.g., relaxation/breathing exercises, self-support group), physical therapies (e.g., physiotherapy, electrostimulation, acupuncture), and complementary alternative therapies	X	X
(v) NQ: type of health care professionals consulted since pain onset and in the months preceding follow-up	X	X
(vi) NQ: continuation of treatment at the pain clinic (yes, no)	X	X
(vii) NQ: patient's disposition after treatment at the pain clinic		X
Patient expectations regarding treatment at the pain clinic		
(i) PQ: expected pain relief *(Pain Relief Scale, 0% = no relief, 100% = complete relief)* [73]	X	
(ii) PQ: patient expected global change regarding functioning level and quality of life *(adapted from the Patient Global Impression of Change Scale) *[28]	X	
Patients' perceived improvement and satisfaction with treatment at the pain clinic		
(i) PQ: patient perception of pain relief *(Pain Relief Scale, 0% = no relief, 100% = complete relief)* [73]		X
(ii) PQ: patient expected global impression of change regarding functioning level and quality of life *(Patient Global Impression of Change Scale)* [28]		X
(iii) PQ: patient satisfaction with treatment *(Satisfaction Scale)* [73]^§^		X
Medical history		
(i) NQ: current and past medical history (type of disorders other than chronic pain)	X	X
(ii) NQ: type of current medication for medical condition	X	X
(iii) PQ: consumption habits (cigarettes, alcohol, illicit drugs)	X	X
(iv) PQ: risk of alcohol and drug abuse/misuse^*ψ*^* (Cage-AID)* [33, 74]	X	
(v) NQ: risk of opioid abuse/misuse^*£*^* (Opioid Risk Tool)* [32, 33]	X	
Demographics		
(i) PQ: date of birth	X	
(ii) PQ: sex	X	
(iii) PQ: ethnic group	X	
(iv) PQ: first language	X	
(v) PQ: education level	X	
(vi) PQ: current living conditions	X	X
(vii) PQ: civil status	X	X
(viii) PQ: current work status	X	X
(ix) PQ: family income	X	X
(x) PQ: main source of income	X	X
(xi) PQ: disability benefits	X	X
(xii) PQ: litigation regarding disability benefits	X	X

NQ, Nurse-administered Questionnaire; PQ, Patient self-administered Questionnaire (PQ).

^*∗*^Follow-up data were collected 6 months after patients' initial visit at the pain clinic. Between November 2008 and March 2012, additional follow-up data were gathered at 12 and 24 months but only in patients who had been not discharged from the pain clinic in the meantime.

^*ψ*^Item not measured after June 2012.

^*£*^Item measured after June 2012.

^§^Patients were informed that no members of the clinical team will have access to their satisfaction ratings regarding the treatments they received at the pain clinic.

**Table 2:** Demographic characteristics of the 6,337 patients enrolled in the Quebec Pain Registry.

	Mean	SD
Age	52.76	14.6

	*n*	%

Sex		
Female	3742	59.1
Male	2595	40.9
Education		
None	23	0.4
Primary	488	7.8
Secondary	2361	37.9
College	1749	28.1
University	1613	25.9
Civil status		
Married/common law	3562	57.1
Single	1458	23.4
Separated/divorced	910	14.6
Widowed	309	4.9
Ethnicity		
Caucasian	5755	92.0
Black descent	162	2.6
Asian	91	1.5
Hispanic	83	1.3
Native	83	1.3
Mixed race	81	1.3
Work status		
Full-time work	1236	19.8
Part-time work	521	8.3
Temporary disability income	1212	19.4
Permanent disability income	1113	17.8
Retired	1299	20.8
Unemployed/laid-off	368	5.9
Homemaker	366	5.9
Student	102	1.6
Volunteer	18	0.3
Other	21	0.3

**Table 3:** Pain-related characteristics of patients enrolled in the Quebec Pain Registry up to December 31, 2013.

	Mean	SD
Average pain intensity in the past 7 days	6.71	2.0
Worst pain intensity in the past 7 days	8.16	1.8
Physical Health-Related QOL (SF-12v2)^*∗*^	29.07	8.9
Mental Health-Related QOL (SF-12v2)^*∗*^	40.48	11.7

	*n*	%

Evidence of neuropathic pain		
(i) No^*ψ*^	1336	23.9
(ii) Yes^*ψ*^	1732	31.0
Mixed evidence^*ψ*^	2511	45.0
Pain duration		
(i) <1 year	702	13.1
(ii) 1 year to <3 years	1201	22.4
(iii) 3 years to <5 years	964	18.0
(iv) 5 years to <10 years	1098	20.4
(v) ≥10 years	1405	26.2
Time elapsed between referral and 1st visit		
(i) <0.5 year	3603	65.0
(ii) 0.5 year to <2 years	1620	29.2
(iii) 2 years to <4 years	297	5.4
(iv) ≥4 years	23	0.4
Pain interference over the past 7 days (BPI score ≥ 7/10)		
(i) General activity	3684	59.1
(ii) Mood	2931	47.0
(iii) Walking ability	2789	44.7
(iv) Normal work	4016	64.4
(v) Relations with other people	2225	35.7
(vi) Sleep	3410	54.7
(vii) Enjoyment of life	2337	37.5
(viii) Self-care	1653	26.5
(ix) Recreational activities	4069	65.2
(x) Social activities	3275	52.5
Depressive symptoms (BDI-I)		
(i) None or minimal (0–9)	1335	21.4
(ii) Mild (10–18)	2167	34.8
(iii) Moderate (19–29)	1786	28.7
(iv) Severe (30–63)	946	15.2

^*∗*^Norm-based scores [44].

^*ψ*^Patients were classified as having nonneuropathic pain if they received a nonneuropathic pain diagnosis from the pain physician and had a score ≤ 3 on the DN4 Questionnaire. A diagnosis of neuropathic pain was defined as a combination of a neuropathic pain diagnosis made by the pain clinician and a score ≥ 4 on the DN4. Patients who had either a neuropathic pain diagnosis from the pain physician or a score ≥ 4 on the DN4 were classified as having mixed evidence of neuropathic pain.

**Table 4:** Type and number of studies for which access to QPR data has been requested up to February 2016.

	Observational studies *n*	Satellite research projects^*∗*^*n*	Feasibility studies *n*	Patient recruitment for external studies *n*	Total *n*
Students					
(i) Undergraduate	1				1
(ii) M.S.	3	—	—	—	3
(iii) Ph.D.	4	2	—	—	6
(iv) Postdoctoral	2	2	—	1	5
(v) Research/clinical fellowship	3	1	—	1	5
Academic researchers	4	1	6	1	11
Industry researchers	4	—	1	1	6
Clinicians	—	—	2	—	2

Total	21	6	9	4	40

^*∗*^Satellite research projects are studies in which QPR data are linked to other data sets (e.g., governmental administrative databases) or to data obtained in the context of a new study collecting variables not contained in the registry (see Section 2.3 – Access Policy).

## References

[1] Bellows B. K., Kuo K.-L., Biltaji E. (2014). Real-world evidence in pain research: a review of data sources. *Journal of Pain and Palliative Care Pharmacotherapy*.

[2] Gliklich R. E., Dreyer N. A., Leavy M. B. (2014). *Registries for Evaluating Patient Outcomes: A User's Guide. Third edition. Two volumes. (Prepared by the Outcome DEcIDE Center [Outcome Sciences, Inc., a Quintiles company] under Contract No. 290 2005 00351 TO7.) AHRQ Publication No. 13(14)-EHC111*.

[3] Dreyer N. A., Garner S. (2009). Registries for robust evidence. *The Journal of the American Medical Association*.

[4] Malmenäs M., Lowton K., Morin I. (2009). Analysis of effectiveness in patient registry data. *ISPOR Connections*.

[5] McQuay H., Moore A. (2007). Utility of clinical trial results for clinical practice. *European Journal of Pain*.

[6] Zaslansky R., Chapman R. C., Meissner W. (2009). Registries for acute pain: will they advance evidence-based practice. *American Pain Society (APS) Bulletin*.

[7] Reid M. C., Bennett D. A., Chen W. G. (2011). Improving the pharmacologic management of pain in older adults: identifying the research gaps and methods to address them. *Pain Medicine*.

[8] Rowbotham M. C., Gilron I., Glazer C. (2013). Can pragmatic trials help us better understand chronic pain and improve treatment?. *Pain*.

[9] Zaslansky R., Rothaug J., Chapman R. C. (2014). PAIN OUT: an international acute pain registry supporting clinicians in decision making and in quality improvement activities. *Journal of Evaluation in Clinical Practice*.

[10] Wolfe F., Michaud K. (2011). The National Data Bank for rheumatic diseases: a multi-registry rheumatic disease data bank. *Rheumatology*.

[11] Smith M. Y., Sobel R. E., Wallace C. A. (2010). Monitoring the long-term safety of therapies for children with juvenile idiopathic arthritis: time for a consolidated patient registry. *Arthritis Care & Research*.

[12] Jarvik J. G., Comstock B. A., Heagerty P. J. (2014). Back pain in seniors: the back pain Outcomes using Longitudinal Data (BOLD) cohort baseline data. *BMC Musculoskeletal Disorders*.

[13] German Research Network on Neuropathic Pain Neuropathic pain data base. 2. http://www.neuro.med.tu-muenchen.de/dfns/projekte/e_periode_3.html.

[14] Moulin D. E., Clark A. J., Gordon A. (2015). Long-term outcome of the management of chronic neuropathic pain: a prospective observational study. *Journal of Pain*.

[15] Nyberg V., Sanne H., Sjölund B. H. (2011). Swedish quality registry for pain rehabilitation: purpose, design, implementation and characteristics of referred patients. *Journal of Rehabilitation Medicine*.

[16] Cook K. F., Buckenmaier C., Gershon R. C. (2014). PASTOR/PROMIS ® pain outcomes system: what does it mean to pain specialists?. *Pain management*.

[17] Griffiths D. P. G., Noon J. M., Campbell F. A., Price C. M. (2003). Clinical governance and chronic pain: towards a practical solution. *Anaesthesia*.

[18] Collaborative Health Outcomes Information Registry (CHOIR) https://choir.stanford.edu.

[19] Solomon D. J., Henry R. C., Hogan J. G., Van Amburg G. H., Taylor J. (1991). Evaluation and implementation of public health registries. *Public Health Reports*.

[20] Gliklich R. E., Dreyer N. A. E. (2007). *Registries for Evaluating Patient Outcomes: A User's Guide. (Prepared by Outcome DEcIDE Center [Outcome Sciences, Inc. dba Outcome] under Contract No. HHSA29020050035I TO1.)*.

[21] Canada S. (2007). *Canadian Community Health Survey (CCHS)*.

[22] Daveluy C. L., Pica L., Audet N. (2000). *Enquête Sociale et de Santé 1998*.

[23] WHO (1978). *International Classification of Diseases*.

[24] CIHI (2001). *The Canadian Enhancement of ICD-10 (International Statistical Classification of Diseases and Related Health Problems, Tenth Revision)*.

[25] Merskey H., Bogduk N. (1994). *Classification of Chronic Pain, Second Edition, IASP Task Force on Taxonomy*.

[26] Bouhassira D., Attal N., Alchaar H. (2005). Comparison of pain syndromes associated with nervous or somatic lesions and development of a new neuropathic pain diagnostic questionnaire (DN4). *Pain*.

[27] Turk D. C., Dworkin R. H., Allen R. R. (2003). Core outcome domains for chronic pain clinical trials: IMMPACT recommendations. *Pain*.

[28] Dworkin R. H., Turk D. C., Farrar J. T. (2005). Core outcome measures for chronic pain clinical trials: IMMPACT recommendations. *Pain*.

[29] Choinière M., Dion D., Peng P. (2010). The Canadian STOP-PAIN project—part 1: who are the patients on the waitlists of multidisciplinary pain treatment facilities?. *Canadian Journal of Anesthesia*.

[30] Kosinski M., Janagap C. C., Gajria K., Schein J. (2007). Psychometric testing and validation of the Chronic Pain Sleep Inventory. *Clinical Therapeutics*.

[31] Brislin R. W., Lonner W. J., Berry J. W. (1986). The wording and translation of research instruments. *Field Methods in Cross-Cultural Research*.

[32] Atluri S. L., Sudarshan G. (2004). Development of a screening tool to detect the risk of inappropriate prescription opioid use in patients with chronic pain. *Pain Physician*.

[33] Webster L. R., Webster R. M. (2005). Predicting aberrant behaviors in opioid-treated patients: preliminary validation of the opioid risk tool. *Pain Medicine*.

[34] Walitt B., Fitzcharles M.-A., Hassett A. L., Katz R. S., Haüser W., Wolfe F. (2011). The longitudinal outcome of fibromyalgia: a study of 1555 patients. *Journal of Rheumatology*.

[35] Choinière M. Pain trajectories in patients treated in tertiary care pain clinics.

[36] Little R. J., Rubin D. B. (1987). *Statistical Analysis with Missing Data*.

[37] Leland Wilkinson and the Task Force on Statistical Inference (1999). Statistical methods in psychology journals: guidelines and explanations. *American Psychologist*.

[38] Kline R. B. (2004). *Beyond Significance Testing*.

[39] Cohen J. (1988). *Statistical Power Analysis for Behavioral Sciences*.

[40] Pearson K. (1904). *On the Theory of Contingency and its Relation to Association and Normal Correlation*.

[41] Cramér H. (1946). *Mathematical Methods of Statistics*.

[42] Ware J. E., Kosinski M., Turner-Bowker D. M., Gandek B. (2004). *How to Score Version 2 of the SF-12 Health Survey*.

[43] Ware J. E., Kosinski M., Keller S. D. (1996). A 12-item short-form health survey: construction of scales and preliminary tests of reliability and validity. *Medical Care*.

[44] Cleeland C. S., Ryan K. M. (1994). Pain assessment: global use of the brief pain inventory. *Annals Academy of Medicine Singapore*.

[45] Larue F., Carlier A. M., Brasseur L., Colleau S. M., Cleeland C. S. Assessing the prevalence and severity of cancer pain in France: the French Brief Pain Inventory.

[46] Tyler E. J., Jensen M. P., Engel J. M., Schwartz L. (2002). The reliability and validity of pain interference measures in persons with cerebral palsy. *Archives of Physical Medicine and Rehabilitation*.

[47] Beck A. T., Ward C. H., Mendelson M., Mock J., Erbaugh J. (1961). An inventory for measuring depression. *Archives of General Psychiatry*.

[48] Gauthier J., Morin C., Thériault F., Lawson J. S. (1982). Adaptation française d'une mesure d'auto-évaluation de l'intensité de la dépression. *Revue Québécoise de Psychologie*.

[49] Cormier S., Lavigne G. L., Choinière M., Rainville P. (2016). Expectations predict chronic pain treatment outcomes. *Pain*.

[50] Giladi H., Choinière M., Fitzcharles M.-A., Ware M. A., Tan X., Shir Y. (2015). Pregabalin for chronic pain: does one medication fit all?. *Current Medical Research and Opinion*.

[51] Giladi H., Scott W., Shir Y., Sullivan M. J. L. (2015). Rates and correlates of unemployment across four common chronic pain diagnostic categories. *Journal of Occupational Rehabilitation*.

[52] Lacasse A., Ware M. A., Bourgault P. (2016). Accuracy of self-reported prescribed analgesic medication use: linkage between the Quebec pain registry and the Quebec administrative prescription claims databases. *Clinical Journal of Pain*.

[53] Lacasse A., Ware M. A., Dorais M., Lanctôt H., Choinière M. (2015). Is the Quebec provincial administrative database a valid source for research on chronic non-cancer pain?. *Pharmacoepidemiology and Drug Safety*.

[54] Pagé M. G., Saïdi H., Ware M. A., Choinière M. (2015). Risk of opioid abuse and biopsychosocial characteristics associated with this risk among chronic pain patients attending a multidisciplinary pain treatment facility. *The Clinical Journal of Pain*.

[55] Scott W., Trost Z., Bernier E., Sullivan M. J. L. (2013). Anger differentially mediates the relationship between perceived injustice and chronic pain outcomes. *Pain*.

[56] Saïdi H. Prevalence of opioid use and characteristics associated with opioid use profile among chronic non-cancer pain patients attending a multidisciplinary pain treatment facility.

[57] Hall G. C., Bryant T. N., Merrett L. K., Price C. (2008). Validation of the quality of The National Pain Database for pain management services in the United Kingdom. *Anaesthesia*.

[58] Cella D., Yount S., Rothrock N. (2007). The Patient-Reported Outcomes Measurement Information System (PROMIS): progress of an NIH roadmap cooperative group during its first two years. *Medical Care*.

[59] Amtmann D., Cook K. F., Jensen M. P. (2010). Development of a PROMIS item bank to measure pain interference. *Pain*.

[60] Hays R. D., Bjorner J. B., Revicki D. A., Spritzer K. L., Cella D. (2009). Development of physical and mental health summary scores from the patient-reported outcomes measurement information system (PROMIS) global items. *Quality of Life Research*.

[61] Cella D., Gershon R., Lai J.-S., Choi S. (2007). The future of outcomes measurement: Item banking, tailored short-forms, and computerized adaptive assessment. *Quality of Life Research*.

[62] Gershon R., Rothrock N. E., Hanrahan R. T., Jansky L. J., Harniss M., Riley W. (2010). The development of a clinical outcomes survey research application: Assessment Center. *Quality of Life Research*.

[63] Saïdi H., Pagé M. G., Ware M. A., Choinière M. Long-term effectiveness of opioids among chronic non-cancer pain patients attending a multidisciplinary pain treatment facility: a Quebec Pain Registry study.

[64] Sturgeon J. A., Darnall B. D., Kao M.-C. J., MacKey S. C. (2015). Physical and psychological correlates of fatigue and physical function: A Collaborative Health Outcomes Information Registry (CHOIR) Study. *The Journal of Pain*.

[65] Sturgeon J. A., Dixon E. A., Darnall B. D., Mackey S. C. (2015). Contributions of physical function and satisfaction with social roles to emotional distress in chronic pain: a Collaborative Health Outcomes Information Registry (CHOIR) study. *Pain*.

[66] Fitzcharles M.-A., Boulos P. (2003). Inaccuracy in the diagnosis of fibromyalgia syndrome: analysis of referrals. *Rheumatology*.

[67] Zaslansky R., Rothaug J., Chapman C. R. (2015). PAIN OUT: the making of an international acute pain registry. *European Journal of Pain*.

[68] Veillette Y., Dion D., Altier N., Choinière M. (2005). The treatment of chronic pain in Québec: a study of hospital-based services offered within anesthesia departments. *Canadian Journal of Anesthesia*.

[69] Peng P., Choiniere M., Dion D. (2007). Challenges in accessing multidisciplinary pain treatment facilities in Canada. *Canadian Journal of Anesthesia*.

[70] Shir Y., Clark A. J., Spanswick C. Novel solutions to a chronic pain problem: improving patients' triaging at tertiary pain clinics.

[71] Sullivan M. J. L., Bishop S. R., Pivik J. (1995). The pain catastrophizing scale: development and validation. *Psychological Assessment*.

[72] French D. J., Noël M., Vigneau F., French J. A., Chantal P., Evans R. T. (2005). L'Échelle de dramatisation face à la douleur PCS-CF Adaptation canadienne en langue française de l'échelle *Pain Catastrophizing Scale*. *Canadian Journal of Behavioural Science*.

[73] Haythornthwaite J. A., Fauerbach J. A., Turk D. C., Melzack R. (2001). Assessment of acute pain, pain relief and patient satisfaction. *Handbook of Pain Assessment*.

[74] Brown R. L., Rounds L. A. (1995). Conjoint screening questionnaires for alcohol and other drug abuse: criterion validity in a primary care practice. *Wisconsin Medical Journal*.

