# Supplementary material for: Development and Implementation of a Registry of Patients Attending Multidisciplinary Pain Treatment Clinics: The Quebec Pain Registry
Source: Pain Res Manag. 2017 Feb 9;2017:8123812. doi: 10.1155/2017/8123812 (PMC5322414; doi:10.1155/2017/8123812)
Supplement: Supplementary file 1 — The first file shows the list of pain diagnoses and their corresponding codes contained in the Quebec Pain Registry (QPR). The pain physicians at the participating sites used this diagnostic grid to record the type(s) of pain the patients suffered from. The second file shows the list of medical interventions and their corresponding codes in the QPR. This grid was used to record the type(s) of medical interventions the patients received. The third file displays the policy to access and use data from the QPR. [file 8123812.f1.pdf]

Supplementary file #1

Diagnostic Grid

How to note the diagnosis

Ex. cervical pain without radicular pain from discal disorder.

General code	Specific code	Etiology code<br>if applicable
**1**	**1.1**	**1.c**

1. CERVICAL PAIN

		SUSPECTED ETIOLOGY
**1.1** Cervical without radicular pain<br>**1.2** Cervical and radicular pain<br>**1.3** Radicular pain only<br>**1.4** Diffuse pain in the cervical region		**1.a** muscular<br>**1.b** facet joint disorder<br>**1.c** disc disorder<br>**1.d** medullary (spinal)<br>**1.e** myofascial (trigger point)<br>**1.f** mechanical<br>**1.g** osteoarthritis<br>**1.h** osteoporosis fracture<br>**1.i** traumatic origin<br>**1.j** following surgery<br>**1.k** following nerve injury<br>**1.l** spinal stenosis<br>**1.m** scoliosis<br>**1.n** spondylolysis/spondylolisthesis<br>**1.o** syringomyelia<br>**1.p** neoplasm<br>**1.q** following cancer treatment (chemotherapy/radiotherapy)<br>**1.r** following HIV and/or its treatment<br>**1.s** infectious<br>**1.t** uncertain origin<br>**1.u** other :
**1.5** Torticollis<br>**1.6** Other :		

2. THORACIC PAIN		
		**SUSPECTED ETIOLOGY**
**2.1** Thoracic without radicular pain<br>**2.2** Thoracic and radicular pain<br>**2.3** Radicular pain only<br>**2.4** Diffuse pain in the thoracic region		**2.a** muscular<br>**2.b** facet joint disorder<br>**2.c** disc disorder<br>**2.d** medullary (spinal)<br>**2.e** myofascial (trigger point)<br>**2.f** mechanical<br>**2.g** osteoarthritis<br>**2.h** osteoporosis fracture<br>**2.i** traumatic origin<br>**2.j** following surgery<br>**2.k** following nerve injury<br>**2.l** spinal stenosis<br>**2.m** scoliosis<br>**2.n** spondylolysis/spondylolisthesis<br>**2.o** syringomyelia<br>**2.p** neoplasm<br>**2.q** following cancer treatment (chemotherapy/radiotherapy)<br>**2.r** following HIV and/or its treatment<br>**2.s** infectious<br>**2.t** uncertain origin<br>**2.u** other :
**2.5** Acute herpes zoster<br>**2.6** Postherpetic neuralgia<br>**2.7** Costochondritis<br>**2.8** Post mastectomy<br>**2.9** Post thoracotomy<br>**2.10** Associated with a neoplasm<br>**2.11** Following cancer treatment (chemotherapy/radiotherapy)<br>**2.12** Following HIV and/or its treatment<br>**2.13** Refractory angina<br>**2.14** Uncertain origin<br>**2.15** Other :		

3. LUMBAR PAIN		
		**SUSPECTED ETIOLOGY**
**3.1** Lumbar without radicular pain<br>**3.2** Lumbar and radicular pain<br>**3.3** Radicular pain only<br>**3.4** Diffuse pain in the lumbar region		**3.a** muscular<br>**3.b** facet joint disorder<br>**3.c** disc disorder<br>**3.d** medullary (spinal)<br>**3.e** myofascial (trigger point)<br>**3.f** mechanical<br>**3.g** osteoarthritis<br>**3.h** osteoporosis fracture<br>**3.i** traumatic origin<br>**3.j** following surgery<br>**3.k** following nerve injury<br>**3.l** spinal stenosis<br>**3.m** scoliosis<br>**3.n** spondylolysis/spondylolisthesis<br>**3.o** syringomyelia<br>**3.p** neoplasm<br>**3.q** following cancer treatment (chemotherapy/radiotherapy)<br>**3.r** following HIV and/or its treatment<br>**3.s** infectious<br>**3.t** sacroiliac<br>**3.u** uncertain origin<br>**3.v** other :
**3.5** Acute herpes zoster<br>**3.6** Postherpetic neuralgia<br>**3.7** Uncertain origin<br>**3.8** Other :		

4. SACRAL PAIN

4.1 Sacroiliitis
4.2 Acute herpes zoster
4.3 Postherpetic neuralgia
4.4 Mechanical pain
4.5 Traumatic origin
4.6 Associated with a neoplasm
4.7 Following cancer treatment (chemotherapy/radiotherapy)
4.8 Following HIV and/or its treatment
4.9 Uncertain origin
4.10 Other :

5. COCCYGEAL PAIN

5.1 Traumatic origin
5.2 Associated with a neoplasm
5.3 Following cancer treatment (chemotherapy/radiotherapy)
5.4 Following HIV and/or its treatment
5.5 Uncertain origin
5.6 Other :

6. ABDOMINAL PAIN

6.1 Acute herpes zoster
6.2 Post herpetic neuralgia
6.3 Post cholecystectomy
6.4 Post herniorrhaphy
6.5 Irritable bowel syndrome
6.6 Chronic pancreatitis
6.7 Crohn’s disease
6.8 Ulcerative colitis
6.9 Following surgery
6.10 Associated with a neoplasm
6.11 Following cancer treatment (chemotherapy/radiotherapy)
6.12 Following HIV and/or its treatment
6.13 Uncertain origin
6.14 Other :

7. PELVIC PAIN

7.1 Endometriosis
7.2 Vulvodynia
7.3 Post herniorrhaphy
7.4 Acute herpes zoster
7.5 Postherpetic neuralgia
7.6 Chronic protatitis
7.7 Interstitial cystitis
7.8 Pudendal neuropathy
7.9 Following surgery
7.10 Associated with a neoplasm
7.11 Following cancer treatment (chemotherapy/radiotherapy)
7.12 Following HIV and/or its treatment
7.13 Uncertain origin
7.14 Other :

8. UPPER LIMB PAIN (LOCAL SYNDROMES)

8.1 Brachial plexopathy
8.2 Bicipital tendinitis
8.3 Subacromial bursitis
8.4 Rotator cuff tear or tendinitis
8.5 Adhesive capsulitis and frozen shoulder
8.6 Lateral epicondylitis
8.7 Medial epitrochleitis
8.8 Cubital tunnel syndrome
8.9 Carpal tunnel syndrome
8.10 Acute herpes zoster
8.11 Postherpetic neuralgia
8.12 Diabetic neuropathy
8.13 Other peripheral neuropathy **8a** mononeuropathy
8b polyneuropathy
8.14 Complex regional pain syndrome
8.15 Stump pain
8.16 Phantom pain
8.17 Myofascial pain (trigger point)
8.18 Traumatic origin
8.19 Following surgery
8.20 Associated with a neoplasm
8.21 Following cancer treatment (chemotherapy/radiotherapy)
8.22 Following HIV and/or its treatment
8.23 Uncertain origin
8.24 Other:

9. LOWER LIMB PAIN (LOCAL SYNDROMES)

9.1 Ischial bursitis
9.2 Trochanteric bursitis
9.3 Osteoarthritis of the hip
9.4 Osteoarthritis of the knee
9.5 Plantar fasciitis
9.6 Sciatica neuralgia
9.7 Obturator neuralgia
9.8 Femoral neuralgia
9.9 Piriformis syndrome
9.10 Plexopathy
9.11 Acute herpes zoster
9.12 Postherpetic neuralgia
9.13 Diabetic neuropathy
9.14 Other peripheral neuropathy
- **9a** mononeuropathy
- **9b** polyneuropathy

9.15 Complex regional pain syndrome
9.16 Postphlebetic pain syndrome
9.17 Stump pain
9.18 Phantom pain
9.19 Myofascial pain (trigger point)
9.20 Restless leg syndrome
9.21 Traumatic origin
9.22 Following surgery
9.23 Associated with a neoplasm
9.24 Following cancer treatment (chemotherapy/radiotherapy)
9.25 Following HIV and/or its treatment
9.26 Uncertain origin
9.27 Other:

10. HEAD AND FACE PAINFUL SYNDROMES

10.1 Migraine
10.2 Headache
- **10a** Tension headache
- **10b** Mixed headache
- **10c** Cluster headache
- **10d** Other type of headache

10.3 Occipital neuralgia (Arnold)
10.4 Trigeminal neuralgia
10.5 Atypical facial neuralgia
10.6 Other myofascial pain (trigger point)
10.7 Acute herpes zoster
10.8 Postherpetic neuralgia
10.9 Temporomandibular joint disorder
10.10 Burning mouth syndrome
10.11 Traumatic origin
10.12 Following dental surgery
10.13 Following other type of surgery
10.14 Associated with neoplasm
10.15 Following cancer treatment (chemotherapy/radiotherapy)
10.16 Following HIV and/or its treatment
10.17 Uncertain origin
10.18 Other:

11. GENERALIZED SYNDROMES

11.1 Fibromyalgia
11.2 Diffuse myofascial pain syndrome
11.3 Rheumatoid arthritis
11.4 Other type of inflammatory arthritis
11.5 Osteoarthritis
11.6 Multiple sclerosis
11.7 Central post-stroke pain (CVA)
11.8 Diabetic polyneuropathy (upper + lower limbs)*
11.9 Polyneuropathies involving more than one limb (upper + lower limbs)**
11.10 Widespread pain of uncertain origin
11.11 Other:

12. PREGNANCY

12.1 Post cesarean pain
12.2 Postpartum pain
12.3 Other pregnancy related pain

13. PAIN RESOLVED

13.1 Pain resolved (no more pain and no more pain medication)

* Do not include the Dx 8.12 and 9.13 if you use the Dx 11.8.
** Do not include the Dx 8.13 and 9.14 if you use the Dx 11.9.

Supplementary file #2

Intervention Grid

1.	Epidural injection	37	Ketamine + versed infusion
2.	Epidural continuous infusion	38	Trigeminal nerve block
3.	Caudal block	39	Ketamine infusion
4.	Intradiscal injection	40	Lidocaine infusion (xylocaïne infusion)
5.	Facet joint block	41	Cortisone infusion
6.	Medial branch block	42	Kadian infusion
7.	Foraminal block	43	Synvisc infiltration
8.	Paravertebral block	44	Biacuplasty
9.	Occipital nerve block	45	Thermocoagulation (Rhizotomy sacroiliac by thermolesion (Sinergy)
10.	Intercostal nerve block	46	Epidural parasagittal
11.	Peripheral nerve block	47	Unknown type
12.	Trigger point injections	48	If other, specifiy
13.	Intra-articular injection		
14.	Intravenous regional blockade		
15.	Injection of botulinum toxin (Botox)		
16.	Intrathecal injection		
17.	Intrathecal continuous infusion		
18.	Spinal cord stimulation		
19.	Sympathetic block (local anesthetic)		
20.	Stellar		
21.	Celiac		
22.	Lumbar		
23.	Impar ganglion		
24.	Sympathectomy (neurolytic agent)		
25.	stellar		
26.	celiac		
27.	lumbar		
28.	Impar ganglion		
29.	Intrathecal neurolysis		
30.	Medial branch neurolysis		
31.	Peripheral neurolysis		
32.	IDET (Intradiscal Electrothermal Therapy)		
33.	Coblation		
34.	Subcicatrical infiltration		
35.	Muscular infiltration		
36.	Lumbar discography		

Supplementary file #3
Access Policy

Quebec Pain Registry – ACCESS POLICY

Access Policy and Use of Data

I. Context

The Quebec Pain Registry (“Registry) is an administrative and research database of chronic pain patients who are treated in tertiary care pain clinics. The Registry uses common demographics, identical clinical descriptions and uniform, validated and standardized measurement tools that may be used in evaluating and promoting evidence-based standards of care. The database is available to facilitate and stimulate clinical pain research for both academia and industry, and to provide a greater understanding of the impact of chronic pain on society. The Registry wishes to allow members of the scientific community access and use of the data to carry out epidemiological, evaluative or clinical research. The Registry can also be used for clinical-administrative purposes by clinicians working at one of the sites participating in the QPR and who are involved in data collection.

II. Objective

The Quebec Pain Registry - Access Policy (QPR – Access Policy) is to oversee applications to be made by researchers or clinicians wishing to use data (QPR - Data Requests). To be authorized, such use must be made in accordance and in compliance with this QPR - Access Policy (“Policy”).

III. Conditions for Use of Registry Data

Access and use of QPR data should made in compliance with the following conditions:

i. Ensure protection of the privacy of the Registry participants. Personal information is confidential. No access to non-anonymized data will be granted except under strict rules approved by the Ethics Committee of the institutions affiliated to the participating sites.

ii. The data are available to persons who carry out research activities, both in the academic sector (e.g.: Universities and Research Institutes) and in the private sector (industry). Data results cannot be sold to another organization. The data are also available to clinicians and policy makers for clinical-administrative purposes.

iii. Before access is granted to the Registry, the application along with the research protocol must obtain approval from the Scientific Review Committee (SRC) by submitting the QPR - Data Request Form. Once approved, any substantive change in the request that the applicant wishes to make must be transmitted to the SRC. Changes in data required will be handled as separate request. Clinicians who wish to access QPR Data must submit the QPR – Data Request Form and obtain approval from the Principal Investigators (PIs) of the QPR (Dr Manon Choinière from the Centre de recherche du Centre hospitalier de l’Université de Montréal and Dr Mark Ware from the Research Institute of the McGill University Health Centre)

All information relating to any access request will be kept confidential unless the applicant provides a written authorization that part of the information can be posted on the Registry website. This information may include name and institution of the applicant, title of the project and non-technical summary of the project.

IV. Scientific Review Committee

The Registry has created a Scientific Review Committee (SRC) and gives this committee the mandate to ensure respect for the QPR - Access Policy with respect to any request and use of data, and to ensure its scientific integrity. Currently, the SRC is composed of the PIs of the Registry and additional members are included when required.

V. Processing a Request

i. The applicant who wishes to have access to QPR data must make a written request to the Registry using the **QPR - Data Request Form** which must be approved and signed by the SRC.

ii. Application for access is considered "under review" until it is determined if the data or reports can be available according to the SRC criteria set in accordance with Registry objectives.

iii. The SRC recognizes that reviews of requests should be conducted in a timely manner.

iv. A decision in favor of the request will be followed by the applicant's review and signature of the "**QPR - Access Agreement**" which is counter-signed by a representative of the SRC.

v. A decision to deny or delay access to the Registry may include suggestions to the applicant of changes that could bring the request for access or use to a favorable decision.

vi. If the request should involve the collection of new variables not contained in the Registry ("Satellite Research Project") , the QPR – Data Request Form signed by both parties along with the research protocol will have to be submitted by the Researcher for review and approval by the Scientific and Ethics Committees of the Centre hospitalier de l'Université de Montréal) and the local institutional committees of the QPR participating sites.

VI. Criteria for Evaluation of a Request

The SRC must evaluate all requests for access with the following criteria:

i. Compliance with the terms and conditions listed in Section III (Conditions for use of data)

ii. The scientific quality of the research project for which the application is associated, including;

   a. The validity of the hypothesis;
   b. The objectives of the research project;
   c. The proposed methodology;
   d. The purpose and intended use of data

VII. Data Access, Use and Delivery

i. Any applicant, whose request for access has been approved, will be provided with the "**QPR - Fee Estimate"** which will detail the costs and expected time frame of output.

i. If agreeable the applicant must complete and sign the standardized "**QPR - Access Agreement**" before any data extraction can begin.

ii. The arrangement for data transfer, prioritization and delivery timing will be finalized between the Registry data management staff and the applicant.

VIII. Publication[1]

i. Applicants who have used or accessed data for research purposes are invited to present and/or publish the results of their project to the scientific community and the general public who may benefit from them. The researcher must state in its publications or presentations that the data used came from the Quebec Pain Registry (See Appendix 1 of the "QPR - Access Agreement" document).

ii. Data provided for clinical-administrative purposes cannot be used for research purposes and published (see Note 1 for definition of publication).

IX. Intellectual Property

The Registry does not claim any intellectual property rights on the results, discoveries, inventions or works that could result from a project research for which Registry data was used.

X. Costs

A non-refundable administration fee is required for any applicant (except clinicians) who submits a request for access. This cost is due when the request is submitted. This fee is to cover review costs and to discourage frivolous requests. It is only charged once per request.

---

[1] Publication is defined as any public presentation of data as any or all of the following mechanisms: slides, abstract, poster, oral presentation/conference, article, or book chapter.

Subsequent fees are required if an applicant is granted access and decides to proceed with use. The fees will be indicated on the **QPR - Fee Estimate** that will be provided to the applicant. These cost recovery fees include paying the cost of preparation and extraction of data and costs to operate the Registry.

XI. Exceptions and Special Circumstances

These policies are intended to serve as a guide to all interested parties on the routine access and use of Registry data. It is recognized that every circumstance that might arise cannot be anticipated. Any requests for consideration of special circumstance or exemption to the stated policies must be presented to the Registry in writing to the SRC who will render judgment.
